# The relationship between COVID-19 stress and test anxiety in art students: the chain mediating roles of intolerance of uncertainty and sleep quality

**DOI:** 10.1186/s12889-024-18684-7

**Published:** 2024-04-25

**Authors:** Ruiying Liu, Qing Qiu, Baojuan Ye

**Affiliations:** 1 Center of Mental Health Education and Research, School of Psychology, School of Education, Jiangxi Normal University, Nanchang, China; 2 School of Intercultural Studies, Post-doctoral Research Station of Psychology, Jiangxi Normal University, Nanchang, China

**Keywords:** COVID-19 stress, Test anxiety, Intolerance of uncertainty, Sleep quality, Art students

## Abstract

**Background:**

The global spread of COVID-19 has brought immense physiological and psychological distress to students, such as test anxiety and poor sleep quality. This study aims to explore the relationship between COVID-19 stress and test anxiety and the mediating roles of intolerance of uncertainty and sleep quality between them.

**Methods:**

A study was conducted in China during the late stage of the pandemic. A total of 936 Chinese art students (age *M* = 18.51, *SD* = 2.11, 46.6% female) completed the Coronavirus Stress Measure (CSM), the 12-item Intolerance of Uncertainty (IUS-12), the Brief Version of the Pittsburgh Sleep Quality Index (B-PSQI), and the Test Anxiety Inventory (TAI). A chain mediation model analysis was conducted to examine the mediating effects of intolerance of uncertainty and sleep quality on the association with COVID-19 stress and test anxiety.

**Results:**

COVID-19 stress was positively associated with test anxiety (*β* = 0.50, *p* < 0.001). The intolerance of uncertainty and sleep quality partially and serially mediated the relationship between COVID-19 stress and test anxiety (*β* = 0.01, 95% CI = 0.01 to 0.02).

**Conclusion:**

These findings suggest that art students’ intolerance of uncertainty and sleep quality partially and serially mediate the relation between COVID-19 stress and test anxiety. The results have significant implications for the intervention and prevention of test anxiety, providing additional evidence for the relationship between COVID-19 stress and test anxiety.

## Introduction

COVID-19 has emerged as a significant health threat, posing a vital threat to the mental and physical well-being of individuals worldwide [1–3]. COVID-19 stress can lead to various physical and psychological problems in individuals such as depression, anxiety, poor sleep quality, and other problems [4, 5]. For the students, the negative effects of COVID-19 stress are mainly related to academic stress and anxiety [6]. The COVID-19 pandemic has brought significant changes to the lives and studies of adolescents. Campus lockdowns, online teaching, and reduced social support have greatly increased students’ academic stress and anxiety [7–9].

Art students refer to specific groups of students defined by the characteristics of their chosen majors. For art students, the admission requirement for the college entrance examination is that they can pass both professional and cultural courses at the same time [10]. Compared to regular academic students, art students in high school have to study more subjects and bear a heavier workload during their three years of high school [11]. In 2014, China began the reform of art college entrance examination, and in recent years, the scores of art students in cultural courses have increased year by year [11]. The Ministry of Education has put forward a series of reform requirements for college art major enrollment in 2019. The admission cultural score line for art-related majors of central colleges and universities cannot be lower than the admission line for the cultural score of the general majors [11]. In addition, the new policy for art college admissions has been gradually implemented since 2021. Universities preferentially admit art students based on the total scores of cultural courses and professional courses, of which cultural scores account for not less than 50% in principle. Compared to previous admission policies, the minimum cultural exam score for art students will be raised [12]. With the reform of China’s art college entrance examination, the admission scores of art students in cultural courses are increasing year by year. The changes in this policy have brought about increased psychological pressure and anxiety among art students who struggle with cultural courses during their studies and exam preparations [11]. Previous studies have also shown that art students report more stress and time spent on academic work than non-art students [10]. In addition, the test anxiety among high school students is more widespread and severe under the pressure of the pandemic [13]. But excessive anxiety not only affects test performance but also harms mental health [14, 15]. Moreover, there are few studies on text anxiety of art student. Given the negative impact of test anxiety on both test performance and mental health, it is essential to understand and focus on test anxiety so that art students can reach their academic potential.

According to Pressure-Cognitive Interaction Theory, individuals make a primary and secondary assessment of stress to assess whether the individual has the ability and resources to cope with the stress. If individuals adapt and can effectively cope with stress, there is no stress response; otherwise, there is a stress response [16]. Therefore, in the face of COVID-19 stress, individuals make a preliminary assessment and secondary assessment. Individuals with high intolerance of uncertainty make negative cognitive interpretation for the COVID-19 stress [17]. When individuals assess that they cannot effectively cope with COVID-19 stress, they may produce physiological and psychological reactions such as poor sleep quality and anxiety. This study examines the impact of COVID-19 stress on test anxiety and explores the serially mediating role of intolerance of uncertainty and sleep quality for this special group of art students.

## The influence of COVID-19 stress on test anxiety

The COVID-19 pandemic has an important impact on an individual’s mental health and well-being, and previous studies have mentioned that COVID-19 stress can increase anxiety, causing various negative effects [18]. COVID-19 stress is defined the degree to which individuals find that life in the COVID-19 pandemic is unpredictable, uncontrollable, or overloaded (that is stressful) [19]. Studies have shown that COVID-19 stress is positively associated with individuals’ levels of anxiety [5, 20]. Test anxiety is a particular form of anxiety characterized by two main components: emotionality and worry [21]. Worry primarily refers to concerns about upcoming evaluations, expectations, and the resulting worries and unease, which involve more cognitive components; and emotionality mainly refers to the accompanying emotional experiences and physical reactions, such as palpitation, nervousness, and other physical symptoms related to autonomic neurological disorders [22].

In the context of the COVID-19 pandemic, the influence of external factors such as COVID-19 stress may exacerbate students’ test anxiety [13]. On the one hand, the COVID-19 pandemic has led to changes in learning styles, resulting in poor learning outcomes for students [9]. In particular, for art students, the study of specialized courses requires a lot of operational exercises, and online learning is not conducive to the study of professional knowledge. In addition, with the change of the policy, the proportion of academic courses in the art exam has increased, which has also increased the academic burden on art students. The culture examination and art examination are life-changing opportunities for many art students. Consequently, art students may experience heightened worries and uneasiness about the culture examination and art examination, which to some extent affects their test anxiety levels. On the other hand, the stress caused by COVID-19 may make individuals feel nervous and anxious [4]. Art students have higher stress levels and poorer mental health than non-art students [10]. And these negative emotional experiences also affect the individual’s test anxiety [23]. For art students, the stress and anxiety caused by COVID-19 may also affect their test anxiety.

## The mediating role of intolerance of uncertainty

Intolerance of uncertainty (IU) is conceptualized as a cognitive bias that influences how individuals perceive, interpret, and respond to uncertain situations at cognitive, emotional, and behavioral levels [17]. During the period of epidemic liberalization, the chance of getting sick with COVID-19 is greatly increased, bringing a lot of uncertainty to the individual’s life, learning and own health [24]. Study have found that the uncertainty and perceived stress of the COVID-19 pandemic are positively correlated with intolerance of uncertainty [25].

In addition, according to the intolerance of uncertainty model, intolerance of uncertainty appears to be an important cognitive element that affects test anxiety [26, 27]. Intolerance of uncertainty is likened to a cognitive bias in which uncertainty and ambiguity are viewed as threatening, and it is proposed to directly lead to worry and anxiety [26]. When faced with uncertain situations, high IU individuals may engage in risky cognitive explanations and experience increased worry and anxiety, which can contribute to heightened test anxiety among students [17, 28, 29]. And previous studies have demonstrated that intolerance of uncertainty is a direct predictor of test anxiety [30, 31]. Studies have shown that student athletes’ intolerance of uncertainty is a significant predictor of test anxiety [32]. Art students, similar to student athletes, face the double pressure of academic and professional courses. Art students report more mental stress and spent more time on academic work than non-art students [10]. Therefore, this study speculates that there is a positive correlation between art students’ intolerance of uncertainty and test anxiety.

## The mediating role of sleep quality

Additionally, sleep quality may serve as another mediating variable worth considering. The COVID-19 pandemic has introduced many unprecedented stressors that negatively affect sleep quality [33, 34]. A larger proportion of the population reported sleep disturbances and poor sleep quality after the COVID-19 outbreak compared to pre-pandemic levels [35, 36]. In addition, compared to older individuals, adolescents with high COVID-19 stress have poorer sleep quality [37]. Art students report increased sleep disturbance and daytime dysfunction, and later chronotype, compared with the non-art students [38]. Studies have shown a positive correlation between COVID-19 stress and sleep quality [34, 37]. Under the influence of COVID-19 stress, art students may experience poor sleep quality.

Based on the transdiagnostic cognitive theory of sleep posits, poor sleep quality can impair individuals’ cognitive functioning such as working memory, concentration, etc., and reduce their ability to manage emotions [39–41]. Individuals with poor sleep quality tend to experience more worry and anxiety in test situations, which may affect their level of test anxiety [42]. Physiologically, sleep-deprived individuals would experience some physical discomfort (increased blood pressure, increased neuro-immunological functioning, increased sympathetic tone) [39]. These physiological discomforts caused by the lack of sleep further exacerbate panic symptoms, nervousness and autonomic dysfunction in individuals and may increase their level of test anxiety [14, 43]. Previous studies have shown a positive correlation between sleep deprivation and higher test anxiety [14].

## The chain mediation effect intolerance of uncertainty and sleep quality

Intolerance of uncertainty is an important factor that affects adolescent mental health, especially regarding sleep problems. Studies have found that adolescents with high intolerance of uncertainty scores have negative cognition in the face of uncertain situations. This increases the arousal of negative emotions which subsequently triggers problems related to sleep [44]. The social uncertainty caused by COVID-19 has disrupted people’s normal sleep time and the quality of sleep has deteriorated [24]. A study conducted during the early COVID-19 pandemic found that uncertainty intolerance plays a crucial role in the relationship between COVID-19 uncertainty and sleep outcomes [24]. Previous studies have shown that intolerance of uncertainty is positively related to sleep quality [44, 45]. Based on these findings, the study further speculates that the intolerance of uncertainty is positively related to sleep quality.

### The current study

Current research on test anxiety mainly focuses on the general student population, with less attention given to the test anxiety of art students. In addition, most studies on the influencing factors of test anxiety focus on family factors and individual internal factors such as coping style and personality characteristics [46, 47]. Few studies explore the effect of COVID-19 stress on individual test anxiety. The epidemic has led to changes in the academic environment and methods, causing anxiety and depression among students [8, 9]. Therefore, the degree and causes of students’ test anxiety during the epidemic period may be different from those in the past. Based on the Pressure-Cognitive Interaction Theory [16], whether COVID-19 stress may affect the art students’ test anxiety depends on the individual’s assessment of COVID-19 stress. Therefore, this study explores the effects of COVID-19 stress on test anxiety of art students from cognitive and physiological perspectives. It enriches the research object of test anxiety and its possible influencing factors.

**Fig. 1:**
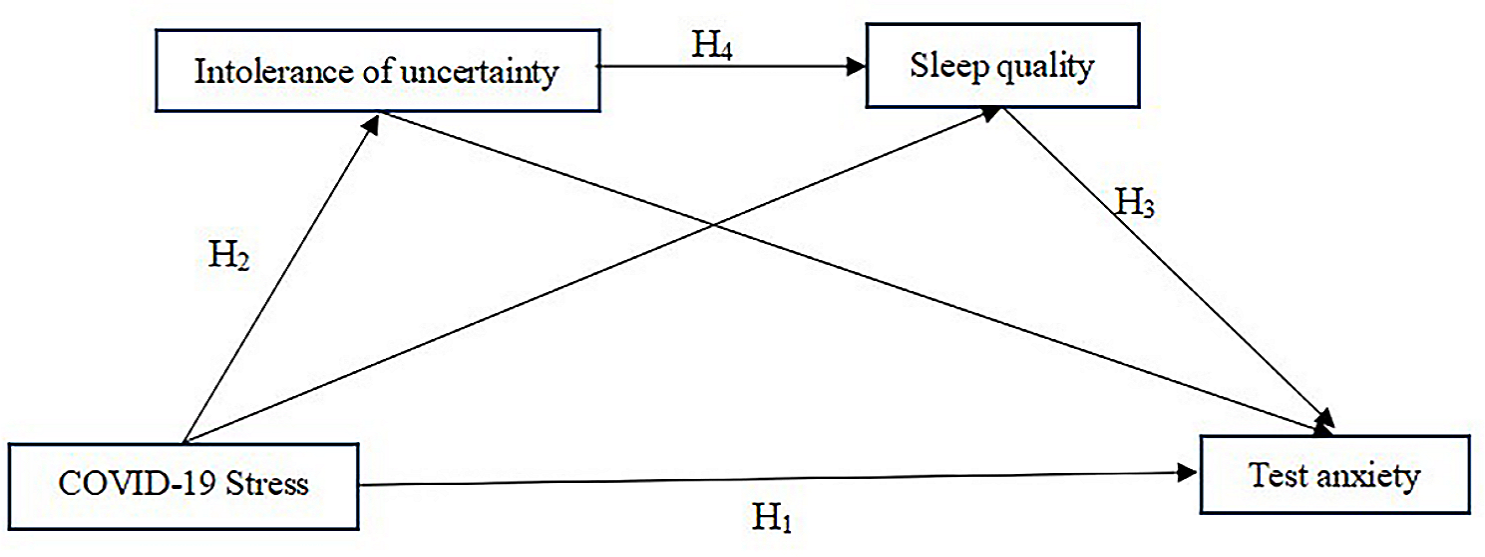
The chain mediation model and hypotheses

Based on the literature review mentioned above and the Pressure-Cognitive Interaction Theory. We conducted a chain mediation model to examine the mediating effects of intolerance of uncertainty and sleep quality on the association with COVID-19 stress and test anxiety. Based on the literature review, we constructed a chain mediation model and proposed the following hypotheses, as shown in Fig. 1

#### Hypothesis 1

COVID-19 stress is positively related to test anxiety of art students.

#### Hypothesis 2

COVID-19 stress can indirectly predict test anxiety of art students through the mediating role of intolerance of uncertainty.

#### Hypothesis 3

COVID-19 stress can indirectly predict test anxiety of art students through the mediating role of sleep quality.

#### Hypothesis 4

COVID-19 stress can indirectly predict test anxiety of art students through the chain mediation role of intolerance of uncertainty and sleep quality.

## Materials and methods

### Participants

A total of 936 senior-year art students who are about to take the Art exam (age *M* = 18.51, *SD* = 2.11, 46.6% female) in China participated in this study. Table 1 shows the detailed demographic characteristics of this study. The consent of the participant was obtained, and the principles of voluntary completion, strict confidentiality and anonymity were emphasized. This study was approved by the Research Ethics Review Committee of Jiangxi Normal University’s School of Psychology.

**Table 1:** Demographic information (*N* = 936)

Characteristics	Categories	*N*	Percentage (%)
Gender	Male	436	46.6
	Female	500	53.4
Only child	Only child	216	23.1
	Non-only child	720	76.9
Father’s level of education	Primary and below	103	11.0
	Junior high school	369	39.4
	Senior high school	261	27.9
	Junior college	90	9.6
	Undergraduate	92	9.8
	Master and above	21	2.2
Mother’s level of education	Primary and below	172	18.4
	Junior high school	371	39.6
	Senior high school	204	21.8
	Junior college	80	8.5
	Undergraduate	89	9.5
	Master and above	20	2.1

### Measures

#### COVID-19 stress

The Chinese version of Coronavirus Stress Measure (CSM) was used to measure COVID-19 stress [20], which was adapted from Arslan et al. [48]. The scale has 5 items (e.g., “How often have you been upset because of the COVID-19 pandemic?”) and each item is rated on a 5-point scale (0 = never, 4 = always). The higher scores indicate higher levels of coronavirus stress. This scale has shown good reliability and validity among Chinese participants [20]. In this study, the α coefficient of the scale was 0.94. Confirmatory factor analysis (CFA) in this study suggested that the one-factor model fit the data well: χ*²/df* = 1.084, RMSEA = 0.009, CFI = 1.000, TLI = 1.000, SRMR = 0.003.

### Intolerance of uncertainty

The Chinese version of the 12-item Intolerance of Uncertainty Scale (IUS-12) [49], adapted from Carleton et al. [50], was utilized to measure Intolerance of uncertainty. The scale consists of 12 items (e.g., “Unforeseen events upset me greatly”) and each item is rated on a 5-point scale (1 = completely inconsistent, 5 = completely consistent). The IUS-12 demonstrated a stable two-factor structure, representing both anxious and avoidance components of intolerance of uncertainty. This scale has demonstrated good reliability and validity among Chinese participants [49]. Higher overall scores indicate higher intolerance of uncertainty. In this study, the α coefficient of the scale was 0.93. Confirmatory factor analysis (CFA) in this study suggested that the two-factor model fit the data well: χ*²/df* = 9.610, RMSEA = 0.087, CFI = 0.955, TLI = 0.937, SRMR = 0.043.

### Sleep quality

Sleep quality was measured by the Brief Version of the Pittsburgh Sleep Quality Index (B-PSQI) [51]. The Brief Version of the Pittsburgh Sleep Quality Index is a brief form of the Pittsburgh Sleep Quality. The Chinese Version of the Pittsburgh Sleep Quality Scale was adapted from Liu et al. [52]. The scale has 6 items (e.g., “When have you usually gone to bed at night?”). Because bedtime and rise time are used to calculate sleep efficiency, the six questions of the B-PSQI yield five scored items. These five items provide a global score ranging from 0 to 15, where higher scores indicate worse sleep quality. This scale has shown good reliability and validity [51]. In this study, the α coefficient of the scale was 0.71. Confirmatory factor analysis (CFA) in this study suggested that the one-factor model fit the data well: χ*²/df* = 2.928, RMSEA = 0.045, CFI = 0.991, TLI = 0.971, SRMR = 0.015.

### Test anxiety

Test Anxiety was measured by the Chinese version of Test Anxiety Inventory (TAI) [53]. The scale has 20 items (e.g., “I felt anxious during the exam”) and each item is rated on a 4-point scale (1 = never, 4 = always). The scale comprises two dimensions: emotionality and worry. Higher overall scores indicate higher levels of test anxiety. This scale has shown good reliability and validity among Chinese participants [54]. In this study, the α coefficient of the scale was 0.95. Confirmatory factor analysis (CFA) in this study suggested that the two-factor model fit the data well: χ*²/df* = 9.610, RMSEA = 0.096, CFI = 0.932, TLI = 0.917, SRMR = 0.038.

### Data analysis

First, we used MPLUS 8.3 to conduct confirmatory factor analysis in order to examine the adequacy of the measurement model for the scales. Model fit was evaluated based on the CFI and TLI values (≥ 0.90), as well as the RMSEA and SRMR values (≤ 0.08) [55]. Second, the descriptive statistics and Pearson correlation were calculated among the study variables using SPSS 25. Finally, the PROCESS macro (Model 6) was applied to examine the mediating effect of intolerance of uncertainty and sleep quality on the relationship between COVID-19 stress and test anxiety. The bootstrap confidence intervals (CIs) were used to determine whether the effects in Model 6 is significantly based on 5000 random samples [56]. An effect is regarded as significant if the CIs do not include zero. All study variables were standardized in Model 6 before data analyses.

## Results

### Common method bias

All data were based on the self-reports of parents on questionnaires. We addressed potential common method bias with Harman’s single-factor analysis [57]. The exploratory factor analysis results showed that there were total of 7 factors with characteristic roots greater than 1. The largest factor explained 37.99% of the variance, with the critical value being less than 40%. Therefore, there are no serious common method biases in this study.

### Descriptive statistics and correlation analysis

Table 2 shows the means, standard deviations, and correlations of the variables. Correlation analysis revealed that COVID-19 stress, intolerance of uncertainty, sleep quality and test anxiety were positively correlated (*p* < 0.001). Most importantly, COVID-19 stress was positively correlated with test anxiety, providing support for Hypothesis 1.

**Table 2:** Means, standard deviations, and correlations of the main study variables

Variables	M	SD	1	2	3
1.COVID-19 Stress	7.62	4.71	-		
2. Intolerance of uncertainty	32.03	9.60	0.42^***^	-	
3. Sleep quality	3.35	2.46	0.25^***^	0.25^***^	-
4. Test anxiety	43.66	11.82	0.51^***^	0.55^***^	0.32^***^

*N =* 936, ^*^p < 0.05, ^**^p < 0.01, ^***^p < 0.001, *M* = Mean, *SD* = Standard Deviation

### The mediating effects of intolerance of uncertainty and sleep quality

**Fig. 2:**
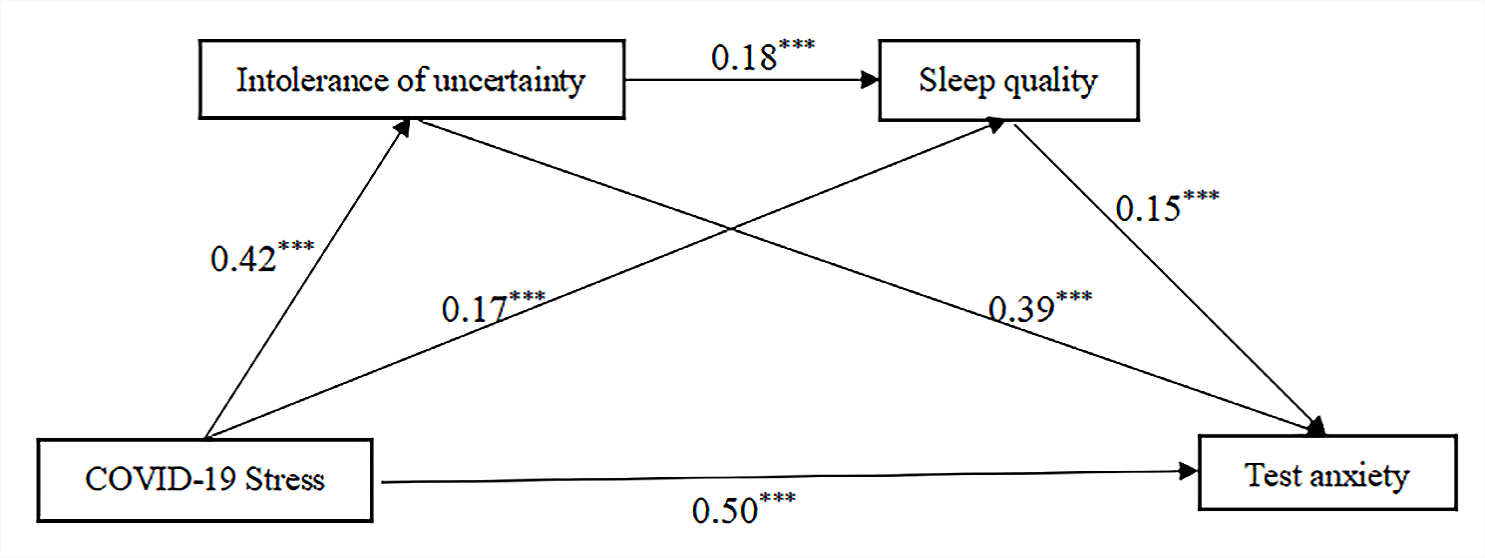
Results of the serial mediation analysis. *Note* The values shown are the standardized coefficients. ^*^P < 0.05, ^**^P < 0.01, ^***^P < 0.001

The results of the serial mediation model are shown in Fig. 2; Table 3. Figure 2 shows the standardized regression coefficients for each path in the model. COVID-19 stress exhibited a significant positive path to intolerance of uncertainty (*β* = 0.42, *t* = 14.22, *p* < 0.001), which in turn had a significant positive path to test anxiety (*β* = 0.39, *t* = 13.65, *p* < 0.001). COVID-19 stress also had a significant positive path to sleep quality (*β* = 0.17, *t* = 4.86, *p* < 0.001), which had a significant positive path to test anxiety (*β* = 0.15, *t* = 5.59, *p* < 0.001). Finally, intolerance of uncertainty had a significant positive path to sleep quality (*β* = 0.18, *t* = 5.26, *p* < 0.001).

**Table 3:** Regression paths of the conceptual model

Variables	Model 1 IU		Model 2 SQ		Model 3 TA	
	*β*	*SE*	*β*	*SE*	*β*	*SE*
Gender	-0.05	0.06	0.00	0.06	-0.02	0.05
Only child	-0.05	0.07	0.02	0.08	0.02	0.06
Father’s level of education	0.10	0.04^*^	-0.09	0.04	-0.01	0.03
Mother’s level of education	-0.02	0.03	0.13^*^	0.04	-0.00	0.03
COVID-19 stress	0.42^***^	0.03	0.17^***^	0.03	0.31^***^	0.03
Intolerance of uncertianty			0.18^***^	0.04	0.39^***^	0.03
Sleep quality					0.15^***^	0.03
*R* ^*2*^	0.19		0.09		0.42	
*F*	44.66^***^		16.10^***^		94.96^***^	

*Notes* IU = intolerance of uncertainty, SQ = sleep quality, TA = test anxiety *N =* 936, ^*^p < 0.05, ^**^p < 0.01, ^***^p < 0.001

Table 4 showed the direct and indirect effects of the model. The direct effect of COVID-19 stress on text anxiety was significant (*β* = 0.31, 95% CI = 0.25 to 0.36). The mediation effect of intolerance of uncertainty on test anxiety was significant (supporting Hypothesis 2) (*β* = 0.16, 95% CI = 0.12 to 0.20). Additionally, the mediation effect of sleep quality on test anxiety was significant (supporting Hypothesis 3) (*β* = 0.02, 95% CI = 0.01 to 0.04). Finally, acting as serial mediators, the indirect effects of intolerance of uncertainty and sleep quality in the relationship between COVID-19 stress and test anxiety were significant (supporting Hypothesis 4) (*β* = 0.01, 95% CI = 0.01 to 0.02). The total indirect effect accounted for 39.05%, with the mediating effect of intolerance of uncertainty at 31.96%, the mediating effect of sleep quality at 4.86%, and the chain mediating effect of intolerance of uncertainty and sleep quality at 2.23%.

**Table 4:** Direct and indirect effects of COVID-19 stress on test anxiety via intolerance of uncertainty and sleep quality

Effect Types	Path	Effect	95% CI		
			Lower	Upper	Effect size
Direct effect	COVID-19 stress→TA	0.3062	0.25	0.36	60.97%
Indirect effect	COVID-19 Stress→IU→TA	0.1605	0.12	0.20	31.96%
	COVID-19 Stress→SQ→TA	0.0244	0.01	0.04	4.86%
	CS→IU→SQ→TA	0.0112	0.01	0.02	2.23%
Total indirect effect		0.1961	0.15	0.24	39.05%
Total effect		0.5022	0.45	0.56	

*Notes* CS = COVID-19 stress, IU = intolerance of uncertainty, SQ = sleep quality, TA = test anxiety, *N =* 936, ^*^p < 0.05, ^**^p < 0.01, ^***^p < 0.001

## Discussion

Although the relationship between COVID-19 stress and anxiety has been extensively studied in recent years, there has been less research on test anxiety for this particular group of art students. According to the norm of TAI scale, a group score greater than 40 points is considered to have a higher score of test anxiety [58]. The results of this study showed that the average score of art students’ test anxiety is 43.66, indicating that art students have a high level of test anxiety. According to TAI assessment standards, 25.4% of the individuals in this study scored more than 50 points and had test anxiety, which was higher than the 14.6% test anxiety detection rate of senior three students in previous study [59]. And previous studies have shown that the level of test anxiety of art students is slightly higher than that of non-art students [60]. According to the results of previous studies, the test anxiety level and test anxiety detection rate of art students are higher than that of non-art students. Therefore, it is necessary to pay attention to the test anxiety of art students.

In this study, these findings suggest that art students’ intolerance of uncertainty and sleep quality partially and serially mediate the relation between COVID-19 stress and test anxiety. The resulting chain mediation model presented here facilitates a better understanding of the relationship between COVID-19 stress and text anxiety of art students. According to the cognitive-phenomenological-transactional model (CPT), stress is caused by both external environment and personal factor [16]. In the face of COVID-19 stress, individuals conduct primary and secondary evaluations, and then make corresponding physiological and psychological responses. Art students with high intolerance of uncertainty tend to give higher evaluations of the harm caused by COVID-19 stress, believing that they are unable to cope COVID-19 stress and the uncertainty of exams. After assessment, individuals may experience physiological reactions such as poor sleep quality, as well as psychological reactions like test anxiety.

### COVID-19 stress and test anxiety

The results showed that COVID-19 stress was positively associated with test anxiety, which is consistent with previous studies [7, 13]. It can be seen that COVID-19 stress, as an external stimulus, has a negative impact on art students’ test anxiety. According to the cognitive-phenomenological-transactional model (CPT), the generation of anxiety emotions depends on an individual’s initial assessment of the event [61]. When art students face COVID-19 stress, they make a preliminary assessment of COVID-19 stress and its risks. If an individual feels unable to handle COVID-19 stress and the examination arrangements, they may experience test anxiety. In addition, China’s college entrance examination is a crucial exam for students. Under the epidemic environment, changes in learning style and reduced social support may also increase test anxiety among art students [8, 9].

### The mediating role of intolerance of uncertainty

The results showed that COVID-19 stress was positively associated with intolerance of uncertainty, and intolerance of uncertainty was positively associated with test anxiety. These results are consistent with previous studies [25, 31]. During data collection, there are still many uncertainties and challenges in the management and control of COVID-19 [25]. And the fear of COVID-19 can also increase an individual’s intolerance of uncertainty [62]. The instability of the epidemic may lead to changes in the timing and location important exams, bringing many uncertain factors to students’ study and life, which contributes to higher levels of COVID-19 stress among individuals and increases their intolerance of uncertainty.

While art students face the dual pressure of the coming cultural examination and professional skill assessments, individuals with high intolerance of uncertainty are more likely to make catastrophic explanations for uncertain events and stressful events. Individuals with high intolerance of uncertainty think that they cannot cope with the possible uncertain events, leading to worry and unease which further intensifies test anxiety [32]. The results found that intolerance of uncertainty played an important mediating role between COVID-19 stress and test anxiety. Cognitive Behavioral Therapy (CBT) can be employed as an effective approach for enhancing individuals’ tolerance for uncertainty [63], thereby effectively reducing test anxiety.

### The mediating role of sleep quality

The results showed that COVID-19 stress was positively associated with sleep quality, and sleep quality was positively associated with test anxiety. These results are consistent with previous studies [14, 36, 64]. The COVID-19 pandemic has exposed people to unpredictable stress, and prior research have shown that perceived stress is a major cause of poorer sleep quality [65]. Stress has been shown to increase the heart rate and increase metabolism, which contributes to lower sleep quality [66]. Therefore, high levels of COVID-19 stress can lead to lower sleep quality and longer sleep latency [34, 67].

Poor sleep quality can affect individuals’ learning ability, memory, concentration and neurobehavioral functioning, and then lead to poor academic performance, and increase individual test anxiety [39, 68]. In addition, poor and inadequate sleep quality is related to reduced positive emotions, cognitive as well as emotional disorders. And individuals with poor sleep quality have weaker emotional regulation ability and are more prone to experiencing anxiety during exams [40, 69]. Relaxation therapy and cognitive-behavioral therapy for insomnia (CBTI) can be used to reduce an individual’s stress level, improve their sleep quality, and help alleviate the production of test anxiety [14, 70].

#### Intolerance of uncertainty and sleep quality as serial mediators

Our results showed that intolerance of uncertainty was positively associated with sleep quality, which is consistent with previous studies [24, 42]. Intolerance of uncertainty has been extensively studied in relation to mental health problems among adolescents, but less research has been conducted on its relationship with sleep quality. Adolescents with higher intolerance of uncertainty showed negative beliefs about uncertain situations and experienced increased arousal of negative emotions [71], which can lead to sleep-related problems [42]. Studies had shown that individuals with high intolerance of uncertainty tend to experience more sleep problems, such as longer sleep latency, decreased sleep duration, and poorer sleep quality [42]. During the COVID-19 pandemic, the chain mediators found here may contribute to reducing art students’ test anxiety from the perspective of intolerance of uncertainty and sleep quality.

This study examined the role of intolerance of uncertainty and sleep quality as a chain mediator between COVID-19 stress and test anxiety. The results showed that the mediating effect of intolerance of uncertainty was the largest, reaching 31.96%, which meant that COVID-19 stress mainly affected test anxiety of art students through intolerance of uncertainty. The mediating effect size of sleep quality was 4.86%, although its mediating effect was not as large as the mediating effect value of intolerant uncertainty, its mediating effect was still significant, indicating that the role of sleep quality in it cannot be ignored.

Furthermore, the chain mediating effect of the intolerance of uncertainty and sleep quality was 2.23%, indicating a significant mediating effect. It is worth noting that the chain mediating effect accounted for a relatively small proportion of the total effect, but this does not mean that the chain mediating effect in this study is meaningless. First, small effect sizes may still have important theoretical implications. Many phenomena in social science are often affected by multiple factors, and social science theories usually only predict whether a certain factor has an impact, but less predict the absolute size of the role played by a certain factor. In this case, if small effects can support the tested theory, then small effects are also of great significance [72, 73]. Secondly, small effect sizes may also have important practical significance, methodologists believe that when small effects may directly or indirectly lead to major results, small effects may accumulate into large effects over time. Even small effects need to be taken seriously [74, 75]. Long-term intolerance of uncertainty and poor sleep quality can affect test anxiety in art students. Therefore, small effects also need to be paid attention to.

#### Implication

It is worth noting that our findings have important academic implications and practical implications. The results showed that the COVID-19 stress positively affected test anxiety of art students, and intolerance of uncertainty and sleep quality played a chain mediating role. It promotes the in-depth understanding of the relationship between COVID-19 stress and test anxiety of art students, and provides important theoretical guidance for schools to carry out students’ mental health education. Schools’ mental health educators can use Cognitive Behavioral Therapy (CBT) to reduce students’ intolerance of uncertainty and improve their sleep quality. This can reduce students’ test anxiety, improve the mental health level of art students, and promote academic development.

## Limitations and further research

Several limitations of the current study need to be considered. Firstly, this study used a cross-sectional design to explore the relationship between COVID-19 stress and test anxiety, which could not explain the causal relationship. Future studies could incorporate longitudinal designs and physiological measurements for in-depth discussion. Secondly, all data in this study were measured by the self-report method, which are subject to socially desirable responding bias. Future studies should collect data from objective sources or multiple informants (e.g., friends, teachers, and family members). Finally, the sample used in this study consisted of Chinese art students, thereby limiting the generalizability of findings across diverse cultures.

## Conclusion

The present study found that COVID-19 stress can aggravate the test anxiety of art students. This effect was partially and serially mediated by intolerance of uncertainty and sleep quality. This study enables us to understand the mechanisms between COVID-19 stress and test anxiety, guiding intervention programs aimed at improving sleep quality and reducing test anxiety.

## Data Availability

The data and materials used in the research are cannot be publicly shared but are available upon request. The data and materials are available from the corresponding author on reasonable request.
